# Developing a data-enabled nudge intervention for childhood antibiotics in primary care: a qualitative study

**DOI:** 10.3399/BJGPO.2024.0032

**Published:** 2025-02-26

**Authors:** Oliver Van Hecke, Aleksandra Borek, Christopher Butler, Sarah Tonkin-Crine

**Affiliations:** 1 Department of Public Health and Primary Care, Ghent University, Ghent, Belgium; 2 Nuffield Department of Primary Care Health Sciences, University of Oxford, Oxford, UK

**Keywords:** antibiotic prescribing, child health, respiratory illness, electronic medical records, primary care

## Abstract

**Background:**

Preschool children (aged ≤5 years) have the highest antibiotic prescribing rate in general practice, mostly for self-limiting acute respiratory tract infections (RTIs). Research from >250 000 UK children suggests that a child’s antibiotic history for RTI may be a good predictor for re-consulting a health professional for the same illness episode and increased clinical workload.

**Aim:**

To develop a data-enabled nudge intervention to optimise antibiotic prescribing for acute RTI based on a child’s antibiotic history in general practice.

**Design & setting:**

Two-phase qualitative study with parents or carers of preschool children and primary care clinicians in England.

**Method:**

In phase 1, through an initial focus group with eight parents or carers and ‘think-aloud’ interviews with 11 clinicians, we co-designed the intervention (computer-screen prompt and personalised consultation leaflet). In phase 2, 13 clinicians used the intervention, integrated into the GP computer software, and shared their feedback through ‘think-aloud’ interviews. Interviews were audio-recorded, transcribed, and analysed thematically.

**Results:**

We co-created a data-driven intervention that automatically integrates a child’s antibiotic history for acute RTI and personalised leaflet into the electronic medical records. We found that parents and clinicians found this intervention, in principle, acceptable and feasible to use in primary care consultations. GP participants reflected on the prompt’s novelty and its usefulness of taking stock of the number of antibiotic prescriptions a child has had in the past year.

**Conclusion:**

Delivering such interventions, integrated into practice workflow, could be efficiently scaled up to promote effective antimicrobial stewardship and reduce unnecessary antibiotic use in primary care. Further research will test this intervention in a future trial.

## How this fits in

Providing automatic, computer-integrated prescribing prompts have led to decreased antibiotic prescribing for acute respiratory tract infections (RTIs) in adults but not in children. Research suggests that preschool children receiving antibiotics for acute RTI in the past year were more likely to re-consult for the same illness episode, increasing clinical workload. Heeding a child’s antibiotic history might be an important cue, which might achieve a change in behaviour without restricting prescribing options. Our data-driven intervention incorporates this antibiotic exposure data as a nudge that prompts clinicians to consider a non-antibiotic strategy for self-limiting acute RTI in preschool children and was on the whole accepted by GPs and parents. If shown to be effective in a future trial, this intervention of efficient design and low cost could be scalable across NHS general practice.

## Introduction

Preschool children (aged ≤5 years) have the highest antibiotic prescribing rate in general practice.^1,2^ The majority of these prescriptions are for self-limiting RTIs where antibiotics do not have any additional clinical benefit,^3–6^ yet at least one in three children are prescribed an antibiotic course for these illnesses.^7^ Previous research has shown the many factors that influence antibiotic prescribing for RTI^8,9^ and, in particular, childhood RTI.^10–14^ Safety remains an overriding principle, and in the context of clinical uncertainty often leads to risk averse overprescribing of antibiotics. A UK primary care database study showed that the proportion of preschool children prescribed an antibiotic course for acute RTI was 45%.^7^ This exposes children to antibiotic side effects and other harms; for example, antibiotic resistance.

There have been many interventions to optimise antibiotic prescribing for acute RTIs. These have often been resource intensive, have many barriers to wider and consistent implementation, or have a modest or transient effect on antibiotic prescribing rates.^15–22^ The most effective interventions are multifaceted and target both patients and clinicians during consultations. Some have shown that providing automatic, computer-integrated prescribing prompts can be helpful.^15,16,23^ These interventions promote current prescribing guidelines, and have led to decreased antibiotic prescribing for RTIs in adults but not in children.^24,25^ This indicates the potential complexity of clinical and shared decision making,^26^ and uncertainty in prescribing for children.^10^ It also signals a need to employ stratified interventions, tailored to specific age groups.^27^

### Developing a ‘nudge’ intervention

The concept of 'nudge' has emerged from behavioural economics to explain and promote improved decision making.^28,29^ Behavioural economics recognises that contextual, psychological, social, and emotional factors influence decision making and that subtle environmental changes, or 'nudges', have the potential to influence people’s behaviour.^30^ A nudge intervention *'is any aspect of the choice architecture that alters people’s behaviour in a predicable way without forbidding any options or significantly changing their economic incentives'*.^29,31^ Put simply, nudge interventions are light-touch behaviour change strategies, providing a subtle way of altering people’s behaviour in a predictable way without forbidding or adding any options or changing incentives. Nudge interventions are simple and low cost, and thus are attractive to managers and policymakers. Nudge interventions have also been applied to antibiotic prescribing.^32^ However, these interventions have mostly come in the form of social norm feedback on prescribing, which focused on clinician peer comparison. The effects on prescribing were often transient and/or labour-intensive.^33,34^

### The importance of a child’s antibiotic history

Research from >250 000 UK children highlighted that those children who had taken ≥2 antibiotic courses for RTIs in the past year had around a 30% greater chance of ‘not responding’ to treatment for future RTIs compared with children who had not taken antibiotics.^7^ Although these ‘antibiotic non-response’ data may relate to suboptimal diagnosis, inappropriate treatment, and/or medicalisation of self-limiting RTIs in children rather than lack of treatment effectiveness, this childhood ‘antibiotic history’ is currently not routinely used during general practice appointments. A snapshot survey of UK parents with preschool children (*n* = 998), showed that 69% of responders would be interested to know their child’s antibiotic history.^35^

A computer prompt that automatically integrates a child’s antibiotic history for RTI might be an important cue that interrupts habitual clinician prescribing, reminding them of antimicrobial stewardship in this age group and creating an opportunity for the clinician to start a discussion with the parent or carer about antibiotics.^14,36^ The ubiquitous use of electronic medical records in UK general practice provides an opportunity to develop a novel data-driven intervention.^27^

We therefore aimed to explore the potential of using a child’s antibiotic history as a data-enabled nudge intervention to optimise antibiotic prescribing for acute RTI in preschool children presenting to general practice.

## Method

The CHRONICLE Study was a two-phase qualitative study involving a semi-structured focus group with parents or carers of preschool children (phase 1) and ‘think-aloud’ interviews with primary care clinicians (phase 1 and 2) to co-produce an automated computer-screen prompt about a child’s antibiotic history and a personalised consultation leaflet. Interviews were audio-recorded and transcribed verbatim. In recognition of their contribution, all participants received a gift voucher.

### Phase 1: co-design of a prototype computer-screen prompt and personalised leaflet

#### Participants and setting

For phase 1, we included parents (or carers) and primary care clinicians.

Eligible parents or carers (aged ≥18 years) included any primary caregiver (for example, parent, adoptive parent, or step-parent) of a preschool child who had had an acute RTI. An RTI was defined as parent-reported symptoms consistent with recent (within 3 months) upper or lower RTI. Parents of children with hospital-acquired infection or serious underlying conditions (for example, cystic fibrosis) were excluded. Participants were sought through several recruitment strategies: community networks; study advertisements through local and national parent support groups (for example, mother and toddler groups); social media; local newspaper and online advertising platforms; and snowball sampling.

Primary care clinicians included any GP or nurse prescriber who prescribes antibiotics in UK general practice using EMIS Health software. Clinicians were recruited through online advertisements in publications targeted at GPs and bulletins of relevant GP organisations (for example, *Pulse*); in locum GP chambers and agencies (for example, National Association of Sessional GPs); and snowballing.

After the initial expression of interest, the participant information sheet and consent form were emailed to participants at least 1 day before interviews. Verbal informed consent was obtained before the start of the interview.

#### Data collection

Phase 1 data were collected remotely and conducted through Microsoft Teams following a semi-structured topic guide. Parents and carers participated together in one focus group first. Clinicians were interviewed individually.

Parents were asked about their views on antibiotics for their children, their perceived benefits and harms, and whether the concept of a child’s antibiotic history might be of interest to parents. Clinician participants were asked about their views on and approach to discussing past antibiotic use with parents, and the potential significance of a child’s antibiotic history. Both sets of participants were asked for feedback on the design and content (including validity and acceptability) of an early prototype computer-screen prompt. Participants were encouraged to give feedback on early draft patient leaflets, which had been designed by an information design specialist. The leaflet content focused on the following three common paediatric infections: acute cough; sore throat; and acute otitis media. The leaflet content was adapted from other readily available information leaflets from major trials^18,27^ and current parent-focused websites; for example, Healthier Together.

The research team initially used document-based prompts in phase 1, with each sheet representing a computer screen shared with participants using the ‘share screen’ function on Microsoft Teams. This was an iterative process retaining a similar outlay for all three leaflets, with prototype computer prompts amended following the feedback from participants, and subsequent participants commenting on the revised versions. The content of the draft prompt and leaflets were further refined in phase 2.

### Phase 2: piloting and refinement of the beta-version computer-screen prompt and leaflet embedded in EMIS Health

From phase 1, a beta-version of the computer-screen prompt was generated together with EMIS software engineers. This involved two aspects:

1. To develop and implement a search algorithm in EMIS Health using Systematized Nomenclature of Medicine Clinical Terms (SNOMED CT) clinical terms. This automatically calculates a child’s antibiotic exposure for acute RTI within the past year and auto-populates the screen prompt. SNOMED CT is a structured clinical vocabulary for use in an electronic health record. The algorithm specifically excluded SNOMED CT clinical terms related to highly specific patient groups in whom specialised antibiotic regimens are recommended for chronic respiratory diseases (for example, cystic fibrosis).^37^
2. To incorporate personalised leaflets within the EMIS software.

In phase 2, we recruited new clinicians using the same approach as in phase 1. Clinicians participated in a face-to-face ‘think-aloud’ interview with the researcher (OVH) to study reactions to the beta-version screen prompt at their work computer. Clinicians activated the prompt at the time of the interview and accessed a ‘dummy’ patient record or a random preschool patient on the EMIS clinical system. Clinicians were asked to ‘think-aloud’ while exploring the prompt and leaflet, that is, to reflect on the features of the prompts, the best time point for the prompt to appear, acceptable leaflet formats, their use during RTI consultations, and say what they thought about each feature and which functions were most or least useful and why.

#### Analysis

Transcripts for both phases were checked for accuracy against the recording and de-identified. Transcripts were analysed (OVH) using thematic analysis aided by specialist software (NVivo version 11) to organise data.^38^ Constant comparison was used to compare and code data across interviews from respective phases, taking an inductive approach. Codes were compared with one another to create categories, grouping similar codes together. This hierarchical coding framework applied to subsequent transcripts. We developed descriptive, cross-cutting themes that helped organise and present the data centred on different topics (for example, feedback on computer-screen prompt) rather than interpretative themes. Agreement on themes, sub-themes, and coding was sought between members of the research team, and 20% of the transcripts were coded by both OVH and AB.

## Results

In phase 1, eight parents (six mothers and two fathers) participated in the focus group in November 2021. Six parents identified as White British and two as British Asian. The focus group lasted 72 minutes. Eleven GPs (five GP partners, four salaried GPs, and two sessional GPs; six male and five female) participated in phase 1 interviews. Interviews lasted on average 32 minutes (range 24–48 minutes).

In phase 2, 13 GPs (seven GP partners, three salaried GPs, two sessional GPs, and one GP trainee; five male and eight female) participated in ‘think-aloud’ interviews. Interviews lasted on average 25 minutes (range 16–35 minutes).

For the purposes of our main research objective, emphasis is given to cross-cutting topics from both phases with original findings illustrated below with quotations.

### Reflections on antibiotics for children with acute RTI

Both sets of participants described the impact of resource constraints in general practice, the advent of remote consultations as the new ‘normal’, and high demand for care.^39^ Parents reported they worried when their child was unwell and said their GPs would spend the bulk of the consultation allaying their worry. Having consulted their GP, parents were often hesitant to start antibiotics citing uncertainty about whether the child really needed antibiotics or concerns about the immediate and long-term antibiotic harms. Parents perceived that GPs did not prescribe many antibiotics to children for acute RTI:

*‘I’ve always thought about the side effects — because obviously GPs don’t prescribe them very often, which I think is good — but when they do prescribe them, I often worry about what sort of side effects my child is going to experience … ’* (Parent 3)

GPs, on the other hand, voiced their frustrations that, despite concerted efforts on their part, children were still receiving antibiotics unnecessarily in other sectors of the healthcare system:

*‘… if you do a full examination, spend a lot of time explaining to* [parents] *why antibiotics will not be useful but harmful, they attend A&E* [accident and emergency]*, 111, or the Urgent Treatment Centre, and eventually when everybody sees these multiple attendances, somebody succumbs and gives antibiotics.’* (Phase 1, GP2)

GPs were aware most RTIs in preschoolers were of viral origin where antibiotics are not indicated. Most perceived that illness severity was more important in the decision-making process than whether the illness was viral or bacterial.

### Perceptions about the usefulness of a child’s antibiotic history

Most parents recognised the potential usefulness of a child’s antibiotic history to personalise the care their child received. For some, this history would be useful as a potential prompt to discuss antibiotic treatment with their GP, if offered. However, not all parents agreed. A few parents thought that a child’s antibiotic history would only be relevant to other (sicker) children receiving many antibiotic courses and not to those children who had none or infrequent antibiotic courses:

*‘I think we’d like to know, but* […] *where antibiotics are very rarely prescribed,* [you] *would probably remember over the last year ... Whether it’s something that needs to be automated through the GP practice, I would probably say no. But that’s just my opinion.’* (Parent 1)

GPs reported being unaware of the published research about antibiotic exposure in preschool children with RTIs and the relatively low number of antibiotic courses needed to impact re-consultation and clinical workload. Some were surprised about how many children receive multiple antibiotic courses for RTIs in a year. A few raised concerns that a child’s antibiotic history may undermine initial prescribing decisions by colleagues and that it does not capture antibiotic prescriptions issued outside routine general practice.

One GP queried the face validity of the prompt. However, most GPs saw the potential value of a prompt to alert the clinician to a child’s antibiotic history, even if providing information relevant for non-respiratory consultations:

*‘I think* [the prompt] *is quite useful. What might be interesting is* [whether the child] *has had five courses of amoxicillin since last year, the average child has 1.3, and that would be quite an interesting thing to show the mum and say look,* [your child has had] *a lot* [of antibiotics] *compared to the average child.’* (Phase 1, GP3)

### Feedback on computer-screen prompt and leaflet

**Figure 1.**
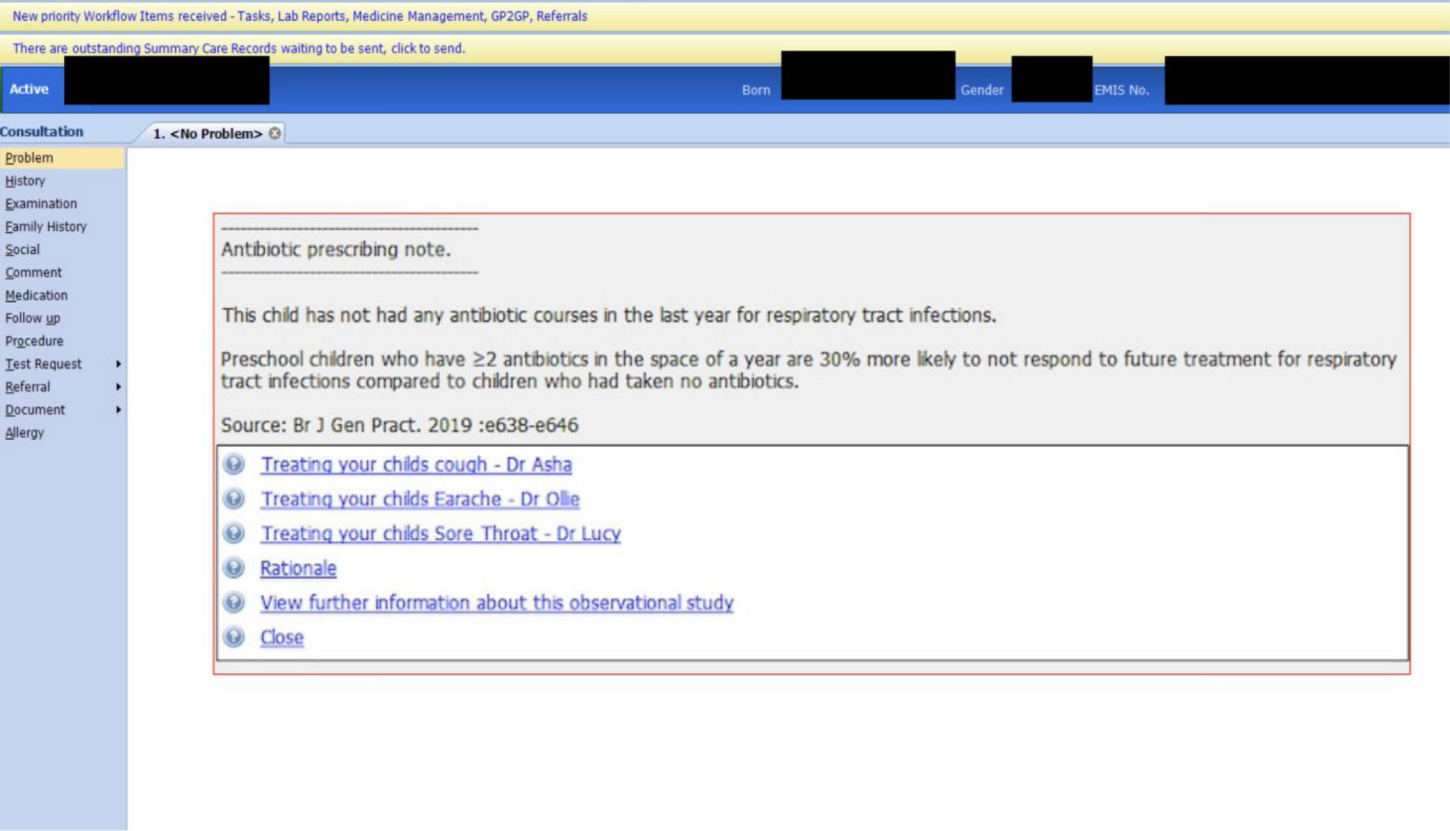
Beta-version computer-screen prompt in phase 2

Phase 1 GPs quickly identified the burden of ‘prompt fatigue' and information overload at the mention of computer-screen prompts. This first impression was echoed by phase 2 GPs when they accessed the beta-version of the prompt (Figure 1):

*‘I’ll be honest with you, there is a lot of prompt fatigue. It just gets to a point where people click buttons to get rid of the prompt.’* (Phase 2, GP1)

However, once phase 2 GPs read the prompt content, most reflected on the prompt’s novelty and its usefulness of taking stock of the number of antibiotic prescriptions a child has had in the past year:

*‘I think it’s good because* [the number of antibiotic courses is] *personalised to the child and then it gives you one key hit you in the face piece of information which is useful ... It doesn’t give you too much, it offers you something useful.’* (Phase 2, GP4)

Some described the screen prompt as a mechanism *‘to engage the brain’* (Phase 1, GP4) to *‘snap you out of that automated routine’* (Phase 2, GP5). Others described the prompt as an *‘extra tool to persuade parents’* (Phase 1, GP1) that antibiotics are mostly not needed for acute RTI in this age group:

*‘I think it is* [...] *useful because you do get set in your ways as a GP.* […] *You have a child with a RTI — you’re in "Autopilot". You’ve done this a thousand times before, and you’re just in that mode … ’* (Phase 1, GP8)

Perceived advantages of the screen prompt included automatically alerting the clinician about a child’s antibiotic history for RTI in the past year and supporting the advice clinicians would routinely give parents. This included providing a useful starting point for discussions with parents about the benefits and harms of antibiotic use:

*‘There’s an expectation by some parents when they bring their poorly child that they’re going to get an antibiotic because that’s what’s happened three times already this year. Knowing this statistic, I think that’s going to be a useful thing to share with the parents.’* (Phase 2, GP7)

All GPs acknowledged that the overarching decision to prescribe an antibiotic was based on their clinical assessment at the time (rather than the prompt itself) but recognised that many RTI in this age group sit in an ‘*equipoise category’* (Phase 2, GP 9), with many competing influences on prescribing:

*‘I think it would influence* [prescribing] *in a sense I would want to give them less* [antibiotics]*, especially if I was on the fence of whether they should have them or not. It would influence me trying to either now or in a future conversation* […] *have a health beliefs conversation about why the child is receiving so many antibiotics.’* (Phase 2, GP13)

GPs broadly envisaged the prompt working in two scenarios. In the first scenario, where a child has had multiple antibiotic courses for acute RTIs and with an unremarkable clinical assessment, the prompt would alert the clinician *‘to try and talk this through with the parents that* [antibiotics] *may not always be appropriate’* (Phase 2, GP10), acknowledging the valid worry that parents have when their child is unwell.

The other scenario was for children who had few antibiotics (none or 1) within the past year for RTI (and an unremarkable clinical examination) where the prompt would now either support those parents *‘who really don’t want to give* [antibiotics] *if they can avoid them and* [those parents] *who are just unsure and just leave it to the clinician’* (Phase 2, GP1).

Parents expressed mixed views and caution in the interpretation of the prototype screen prompt. Some perceived potential value in challenging the commonly held belief that antibiotics are needed for childhood RTI. Other parents voiced concerns about how the screen prompt might be misinterpreted as blaming parents for having a sickly child or ‘wrongly’ consulting the GP, and inadvertently causing more anxiety in already worried parents:

*‘I think there’s the worry that the parents would feel like they’re being* [judged]*. The child has had antibiotics quite a few times and this prompt comes up, whether that’s some sort of reflection on them as bad parents.'* (Parent 5)

However, GPs appeared not overly concerned about parents misinterpreting the screen prompt provided there was careful communication between parents and clinician. Some GPs perceived that this new personalised information might positively influence parents' health-seeking behaviour.

### Timing of screen prompt

Both sets of GP participants recognised the screen prompt would need to appear at the start of the consultation (that is, on accessing the child’s medical records) rather than at the time of prescribing or entering consultation codes. That the prompt might appear for non-respiratory ailments was acceptable as there was a *‘50:50 chance’* (Phase 2, GP6) that the consultation would relate to an RTI in preschoolers:

*‘I think it’s important to have this before the consultation starts, rather than when you click “Prescribe”, and then* [the prompt] *pops up because, by that point, you’ve almost always made up your mind.’* (Phase 1, GP9)

### Limitations of computer-screen prompt

Phase 2 GPs underlined a few limitations associated with the prompt. The main limitation was the inability to capture all antibiotic prescribing data for RTI in the community; for example, out-of-hours setting. Likewise, the prompt would not be seen by GPs who only open a new consultation after the patient has left the room. Clinicians recognised that the prompt may only have impact in consultations where there have been multiple antibiotic courses prescribed within a year. A few were concerned that less experienced prescribers might be swayed not to prescribe when clinically indicated.

### Reflections on leaflets about common RTI

Parents reported that their GPs would rarely give them leaflets following a consultation, which phase 1 GPs confirmed. Instead, GPs preferred to signpost patients to specific websites for further information. However, many parents were keen on a personalised leaflet as this was seen as giving parents *‘something to refer back to if the child became unwell again’* (Parent 3). This could also be a paperless version of the leaflet sent to their mobile phone. In terms of content, parents wanted to have specific details about the illness itself, its anticipated trajectory, (any) additional benefit of taking an antibiotic, alternative medication(s) to try at home, and symptoms and signs to look out for (visual traffic-light red flags).

**Figure 2.**
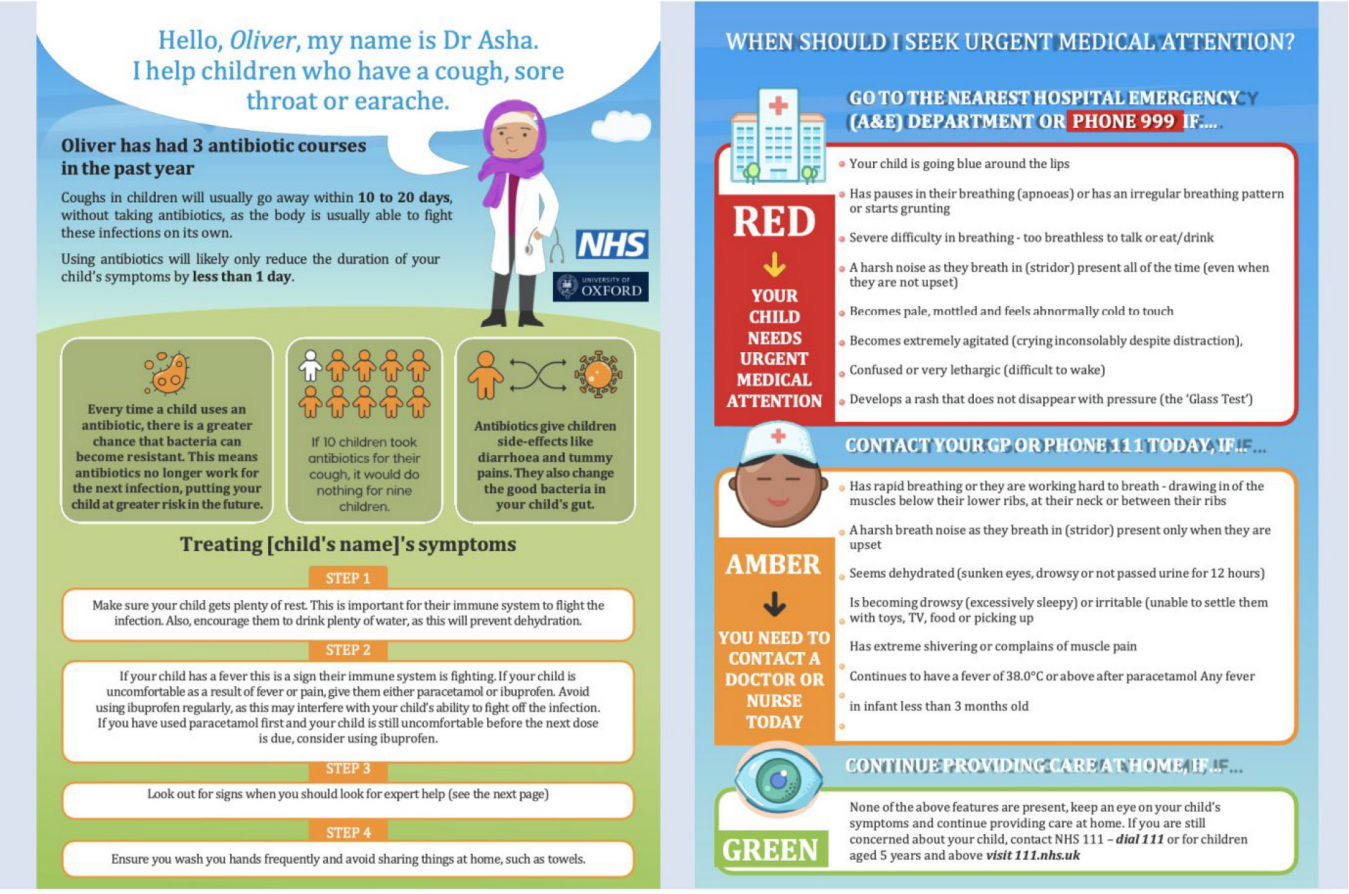
Example leaflet developed in the study for acute cough

The leaflets embedded within the prompt (for example, Figure 2) were well received overall by phase 2 GPs. GP participants commented on the ease of access and content validity congruent with advice they would give to parents about the expected illness course, the benefits and harms of taking antibiotics, self-care options, and safety-netting advice. They welcomed the leaflet’s colourful graphics and the personalised information (for example, inclusion of the child’s name and number of antibiotics prescribed for acute RTI in the past year) making it appealing to read. GPs particularly welcomed the general safety-netting advice, as this was often difficult to do comprehensively and without the reassurance that the parent had fully understood this advice.

## Discussion

### Summary

We have co-produced a data-driven intervention, embedded in the electronic medical records, which integrates a child’s antibiotic history for acute RTI and summarises this in a personalised leaflet. Most parents and GPs saw the potential benefit of the proposed intervention. GPs had not appreciated the relatively low number of antibiotic courses needed to impact their clinical workload by encouraging re-consultations for self-limiting RTI.

### Strengths and limitations

Using a bottom-up approach and transdisciplinary team (clinicians, parents, software engineers, information design specialists, and researchers), we have produced a stratified, data-driven intervention tailored to preschool children with acute RTI. The intervention is of efficient design, low cost, and potentially scalable across NHS general practice.

Using the principles of behavioural economics^30^ and integrating personalised data into a succinct screen prompt is innovative. The prompt does not restrict prescribing decisions. We were specifically mindful not to encroach on clinical autonomy as part of the prompt content and purposefully did not direct clinicians to guidelines.^40^ In view of the enormous variation at which point of the consultation clinicians enter information into the medical records, prompts were activated at the point of accessing the child’s medical records. We have adapted scientific information into a concise, parent-friendly leaflet, personalised to the child, which can be slotted into existing GP text-messaging software.

We accept that there are limitations. Methodologically there is a limitation of interview methods where participants may filter what they disclose to the researcher by giving socially desirable answers. However, both parents and GPs were happy to speak freely about potential negative aspects of screen prompts within the consultation. We would have liked to include other non-GP clinicians (for example, physician associates and nurse prescribers) to glean their feedback. Likewise, we recognise the shortcomings of including one parent focus group of eight parents or carers. However, this focus group gave us early insight into parents' views on a child’s antibiotic history before approaching clinicians as the target group for this intervention. The voluntary nature of participation in the study and the use of convenience sampling may have led to an unrepresentative participant sample taking part, which may have led to more favourable responses.

There are limitations in the design and functionality of the prompt within EMIS Health software as the primary purpose of the software is for medical note-keeping and not research. It is not possible to capture all community antibiotic prescribing data for childhood RTI through EMIS. The personalised leaflets are currently only in English.

We asked participants about a hypothetical scenario where such a prompt might be used during the consultation and not their feedback after ‘real’ GP consultations. This and the proposed mechanisms (see Supplementary Figure S1) will be important to clarify in the next phase of the research.

### Comparison with existing literature

Electronic screen prompts in the field of antibiotic stewardship are not new.^16,24,33^ Most prompts have centred around increasing clinicians’ adherence to antibiotic prescribing guidelines, often as part of a multimodal intervention. However, in these studies, clinicians often had to navigate through informative yet complex prompts detailing: a summary of antibiotic prescribing recommendations, a printable patient information sheet, a summary of research evidence concerning no antibiotic or delayed antibiotic prescribing strategies, information on the definite indications for antibiotic prescription, as well as information and evidence on the risks of not prescribing. The uptake and routine use of such prompts were mixed even in controlled trial settings.^40^ Trial GP participants reported that such prompts were often not used (or used rarely) owing to the limited consultation time to read the prompts. Some GPs reported not needing them as they claimed that they were already following the advice recommended in the guidelines.^40^ In the above trials, prompts were activated on entering consultation codes rather than at the point of accessing the child’s medical records.

There are good existing parent-facing leaflets informed by rigorous research and addressing common concerns held by parents, supporting their capability to care for their child at home and when to seek help.^41,42^ Although used in mostly effective multifaceted trials in the UK,^18,27,42,43^ one could argue that some leaflets were too lengthy for busy parents to read (for example, 8-page booklet on RTI in children), not sufficiently specific to the presenting type of RTI illness, or the degree of personalisation did not go beyond that of the name of the clinician and child. This hampers wider and consistent implementation. In contrast, our 2-page leaflets were personalised, included the child’s antibiotic history for RTI, gave specific information to the diagnosis, and could be incorporated into existing GP text-messaging software.

### Implications for research and practice

Exposure to antibiotics has important clinically relevant implications especially in young children^44^ and potentially predisposes them to higher rates of RTI.^45,46^ Clinicians need to be cautious. The decision to prescribe an antibiotic needs to be balanced with the likelihood that repeated antibiotic exposure early in life has consequences for the child in terms of their heath but also impacts on clinicians’ workload.^7^ Likewise, further research is needed to address the general public’s misunderstanding of antibiotic resistance and that (even) using antibiotics a few times a year for mostly self-limiting RTI is harmful.^47,48^ One option might be to use such an electronic prompt to support this message with specific reference to childhood acute RTIs.

This study illustrates the potential value of using electronic health records data to provide clinicians and parents with useful and succinct personalised information about a child’s antibiotic history. We have taken on board participants’ feedback to create an electronic prompt that fits within the consultation flow and does not impinge on clinical autonomy. The prompt offers a ‘light-touch’ behaviour change strategy, providing a subtle way of altering people’s behaviour. Both participant groups welcomed the embedded personalised leaflet (that is, when the leaflet is accessed, an entry is automatically entered within the medical records).

For policymakers, such low cost, efficient interventions can be scaled up nationally if shown to be effective. Future research will aim to test this intervention in a future trial.

## Data

The dataset relied on in this article is available from the corresponding author on reasonable request.
